# Nonlinear and sex-specific effects of V1aR deletion on juvenile social behavior

**DOI:** 10.3389/fendo.2026.1901426

**Published:** 2026-09-08

**Authors:** D Aspesi, H Vu, I Rivers, MC Stoehr, KL Huhman, HE Albers

**Affiliations:** Neuroscience Institute and Center for Behavioral Neuroscience, Georgia State University, Atlanta, GA, United States

**Keywords:** arginine vasopressin, sex differences, social avoidance, social interaction, social recognition, Syrian hamster

## Abstract

**Introduction:**

Social behaviors emerge during juvenile development and require the integration of social motivation, recognition, and context-appropriate responses. Arginine vasopressin signaling through the V1a receptor (V1aR) has been implicated in multiple aspects of social behavior, yet its role across distinct behavioral domains, developmental stages, and sexes remains poorly understood.

**Methods:**

We used a CRISPR/Cas9-generated V1aR knockout model in Syrian hamsters to examine the effects of V1aR disruption on social interaction, social recognition, and social avoidance in juvenile males and females. Hamsters were tested during the peripubertal period (postnatal days 35–36), prior to the onset of stable estrous cyclicity in females.

**Results:**

In a dyadic social interaction assay, V1aR deletion increased social investigation, particularly anogenital investigation, in knockout animals, whereas heterozygous males exhibited reduced locomotor activity and increased self-directed behaviors. In a social odor recognition task, heterozygous animals failed to discriminate between familiar and novel conspecific odors at a 20-min interval, while wild-type and knockout animals showed intact recognition. No odor discrimination or genotype-dependent differences were observed at a 24-h interval, indicating limited evidence of long-term retention across groups. Following social defeat, heterozygous males displayed increased social avoidance, spending less time near an aggressor and more time in the avoidance zone compared with other groups.

**Discussion:**

Together, these findings reveal domain-specific and nonlinear effects of V1aR disruption on juvenile social behavior, with heterozygous animals exhibiting the most pronounced alterations. These results suggest that V1aR contributes to social behavior in a nonlinear, genotype-dependent, and context-dependent manner and highlight the importance of developmental stage and sex in shaping vasopressin-dependent social processes.

## 1 Introduction

Social behaviors emerge early in development and are essential for survival across species, supporting processes such as communication, reproduction, and the establishment of social hierarchies. These behaviors rely on distributed neural circuits that integrate sensory, motivational, and cognitive information to guide appropriate responses to conspecifics (1, 2). Core components of social behavior include social interaction, social recognition, and the ability to appropriately approach or avoid others depending on context. Disruptions in these processes are a hallmark of several neurodevelopmental and psychiatric disorders, including autism spectrum disorders, schizophrenia, and anxiety-related conditions (3–5), and many, if not most of these disorders are observed more often in one sex (6–8). Understanding the neurobiological mechanisms that regulate social behavior in males and females is therefore critical for both basic neuroscience and translational research.

During the juvenile period, social interactions such as play, investigation, and responses to social challenge contribute to the maturation of neural circuits that support adult social behavior. In many rodent species, including Syrian hamsters, this stage is characterized by elevated levels of social interaction and play, which peak during early adolescence and decline thereafter (9–13). Experimental manipulations disrupting play or social experience during this period lead to long-lasting alterations in social, emotional, and cognitive function, highlighting the importance of this developmental stage (14–16). These processes occur in both males and females, yet sex differences in social behavior often emerge during development, underscoring the importance of examining both sexes within the same experimental framework. Thus, investigating the neurobiological substrates of juvenile social behavior provides key insights into how adult social phenotypes are shaped.

Among the neurochemical systems that regulate social behavior, the neuropeptide arginine-vasopressin (AVP) plays a central role. This system forms part of a conserved “social behavior neural network” that integrates sensory, motivational, and emotional components of social interactions (17, 18). Within this network, AVP signaling through the vasopressin 1a receptor (V1aR) has been implicated in multiple domains of social behavior, including social recognition, aggression, and social communication (19–21). A large body of work demonstrates that V1aR is critical for social recognition, the ability to discriminate between familiar and unfamiliar conspecifics. In mice, genetic deletion of V1aR produces profound impairments in social recognition memory, effects that can be rescued by restoring receptor expression in specific brain regions such as the lateral septum (22, 23). These findings have led to the view that AVP–V1aR signaling is a key regulator of social memory circuits, particularly within olfactory and limbic pathways (17, 24). Beyond recognition, V1aR signaling has also been implicated in modulating social approach and avoidance behaviors, including aggression and anxiety-like responses, suggesting a broader role in assigning valence to social stimuli (25, 26). Importantly, the vasopressin system exhibits pronounced sex differences in both anatomy and function. Males typically show denser AVP projections in regions such as the lateral septum and bed nucleus of the stria terminalis, as well as sex-specific patterns of receptor expression (27, 28). These differences have been linked to sex-specific regulation of social behaviors, particularly in adulthood. Importantly, increasing evidence suggests that AVP signaling during juvenile and adolescent development has translational relevance. For example, alterations in AVP-dependent mechanisms following childhood stress have been linked to persistent impairments in social functioning, highlighting the importance of this neuropeptide system during critical developmental periods (29). Understanding how V1aR contributes to social behavior during the peripubertal period may therefore provide insight into developmental mechanisms underlying social dysfunction in neuropsychiatric disorders.

Despite much progress, several important gaps remain. First, most studies have focused on adult animals, while less is known about how AVP signaling contributes to social behavior during juvenile development, a period preceding the full maturation of gonadal hormone systems and stable estrous cyclicity. Second, many studies have examined males and females separately or focused predominantly on one sex, limiting our understanding of sex-dependent effects. Studying both males and females during development is therefore essential to determine whether sex differences are already present or emerge later in life. Third, V1aR function is often studied within a single behavioral domain, making it difficult to determine whether it acts as a common regulator of social behavior or differentially modulates distinct components such as social interaction, recognition, and avoidance. Importantly, much of our current understanding of V1aR function derives from studies in mice and rats, species that differ substantially in their social organization and behavioral repertoires from other rodents. Syrian hamsters (*Mesocricetus auratus*), in contrast, provide a valuable model for addressing these questions. Unlike many commonly used laboratory rodents, both male and female hamsters exhibit strong social discrimination, robust territorial aggression, and context-dependent social approach and avoidance behaviors (17, 30). Prior pharmacological studies in hamsters have demonstrated that AVP signaling regulates aggression and social communication, but the specific contribution of V1aR to multiple domains of social behavior within this species remains incompletely understood (25, 31). Moreover, social behaviors in hamsters undergo significant changes during the juvenile and peripubertal period, making this species particularly well suited for examining developmental aspects of social behavior.

Recent advances in CRISPR/Cas9 genome editing have enabled the generation of V1aR knockout (KO) hamsters, providing a powerful tool to directly test the causal role of this receptor in social behavior. Notably, initial studies using this model have revealed unexpected and sometimes paradoxical effects, with V1aR deletion producing context-dependent changes in social interactions that do not fully align with findings from traditional mouse models (32). These findings raise the possibility that V1aR function may not follow a simple linear relationship with behavior but instead may exhibit dosage-dependent or nonlinear effects. Despite these advances, no study to date has systematically examined how V1aR influences multiple, functionally distinct domains of social behavior within the same individuals, including social interaction, social recognition, and social avoidance. These domains rely on partially overlapping but dissociable neural mechanisms, and their integration is critical for adaptive social functioning. Understanding whether V1aR acts as a common regulator across these domains or differentially modulates specific components of social behavior remains an important unresolved question.

In the present study, we used a CRISPR/Cas9-mediated V1aR KO model to examine the role of V1aR in multiple domains of social behavior in juvenile male and female Syrian hamsters. Animals were tested during the peripubertal period (PND35–36), prior to the onset of stable estrous cyclicity in females, allowing us to assess social behavior in a developmentally comparable state across sexes. We evaluated social interaction, social recognition, and social avoidance within the same experimental framework to determine whether V1aR signaling exerts a unified influence on social behavior or differentially regulates specific domains. Based on prior work, we hypothesized that V1aR disruption would impair social recognition and alter social approach–avoidance behavior. However, given recent evidence for paradoxical effects of V1aR deletion, we also considered the possibility that partial and complete loss of V1aR might produce distinct, potentially nonlinear behavioral outcomes.

## 2 Material and methods

### 2.1 Subjects

Gene-edited Syrian hamsters (*Mesocricetus auratus*) were generated as previously described (32). A single founder female was bred to wildtype (WT) sires to produce two litters, and offspring were subsequently outbred to WT animals for two additional generations. Male and female heterozygotes (Het) from the F3 generation were then crossed to produce WT, Het, and KO offspring. All subjects used for data collection were derived from the F3 generation or their descendants. WT controls were generated from Het × Het or Het × WT crossings whenever possible. Additional WT animals were obtained either from WT-only litters within the colony or purchased from Charles River Laboratories (Wilmington, MA). The CRISPR/Cas9 strategy used in this study produced a germline (global) deletion of the *Avpr1a* gene, resulting in loss of V1aR expression in both central and peripheral tissues (32).

Animals of both sexes were weaned at postnatal day (PND) 21 and tested at PND35–36. All hamsters were genotyped at weaning using DNA obtained from ear biopsies and analyzed by real-time PCR using allele-specific probes targeting the *Avpr1a* mutation (Transnetyx, Cordova, TN; see 32 for assay validation). Animals were classified as wild-type (WT), heterozygous (Het), or knockout (KO) and assigned to the corresponding experimental group. Separate cohorts were used for each behavioral paradigm; therefore, each hamster participated in only one behavioral assay and no longitudinal testing was performed. After weaning, hamsters were housed in same-sex groups of three to five animals. Experimental animals for the behavioral studies were obtained from multiple independent litters. To minimize potential litter effects, no more than two to three animals of each sex from a given litter were included in each behavioral experiment. Female hamsters were tested prior to the typical onset of stable estrous cyclicity in this species (~PND40–45). To assess reproductive status, females were monitored daily for external indicators of estrous cycling (e.g., post-estrus vaginal discharge) for four days prior to behavioral testing (33). No animals exhibited evidence of estrous cycles during this period, indicating that subjects were in a pre- or early peripubertal state. All hamsters were housed on a 14:10 h light/dark cycle (as is standard in this species to achieve gonadal patency in adults; lights off at 10:00 h) with food and water available *ad libitum*. Animals were maintained in polycarbonate cages (24 × 33 × 20 cm) containing phytoestrogen-free corncob bedding and nesting material. Experimental subjects were handled daily for 3–5 days prior to testing to habituate them to the experimenter.

For the social interaction test, each subject was paired with a same-age, same-sex, weight-matched, group-housed stimulus animal. For social recognition testing, adult scent donor hamsters (PND ~60) were single-housed for at least 7 days prior to scent collection. For social avoidance testing, resident aggressors (RAs) were larger adult hamsters that were single-housed for at least one month and reliably displayed aggression toward same-sex intruders in their home cage. Male RAs were used for male subjects. For female subjects, ovariectomized female RAs were used to avoid variability in aggression associated with the estrous cycle. Stimulus animals, scent donors, and RAs were purchased from Charles River Laboratories and were not part of the genetically modified colony.

All procedures were approved by Georgia State University’s Institutional Animal Care and Use Committee and conformed to the NIH Guide for the Care and Use of Laboratory Animals (34), as well as the Animal Welfare Act Code of Federal Regulations. The Georgia State University animal care and use program is fully accredited by AAALAC International.

### 2.2 Behavior

**Figure 1:**
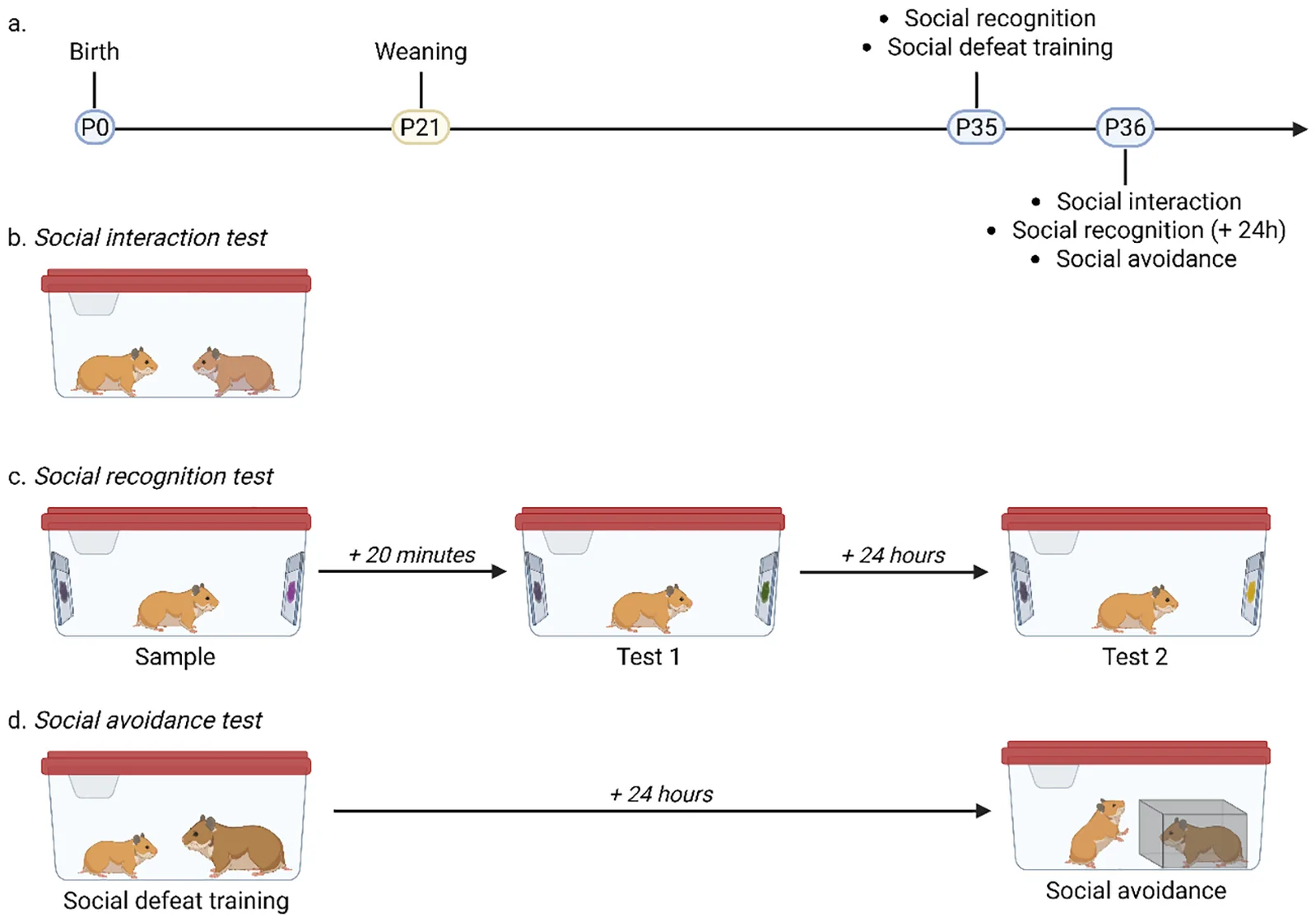
Experimental timeline and behavioral paradigms used to assess social behavior in juvenile Syrian hamsters. **(A)** Timeline of experimental procedures. Animals were born at postnatal day (P)0, weaned at P21, and tested during the juvenile period (P35–P36). Social recognition and social defeat training were conducted at P35, followed by social interaction, social recognition (24 h), and social avoidance testing at P36. **(B)** Social interaction test: subjects were allowed to freely interact with an unfamiliar conspecific in a neutral arena for 5 min. **(C)** Social recognition test: animals were first exposed to social odors during the sample phase, followed by a test phase 20 min later (Test 1) and a second test 24 h later (Test 2), in which investigation of familiar versus novel odors was assessed. Dark and light purple stains on slides represent odors from conspecifics during the sample phase. During the test phases, one odor remained (dark purple; *familiar*), whereas the other was replaced with a *novel* odor (green or yellow stain). **(D)** Social avoidance test: animals underwent 15 min of social defeat training and were tested 24 h later for avoidance behavior toward a confined resident aggressor.

All behavioral testing was conducted during the first three hours of the dark phase to minimize circadian variability and took place in a room with red dim lights. On the day of behavioral testing, animals were transported to the behavioral suite and were given at least 30 min to habituate to the environment. All behavioral tests were videorecorded from above and scored later by a trained experimenter blind to experimental conditions. Schematics of all behavioral paradigms are reported in **Figure 1**.

#### 2.2.1 Social interaction test

Social behavior was assessed in a 5-min dyadic social interaction test conducted in a clean, novel cage (23 × 43 × 20 cm). Each subject was paired with a same-age, same-sex, weight-matched stimulus animal, and the behaviors displayed by the experimental subjects were quantified. All behavioral measures are described in **Table 1**. Examples of behaviors scored include social (including social play and social investigation), nonsocial (e.g., locomotor/exploratory activity), aggression, flank marking, which is defined as rubbing of the flank gland region against cage surfaces, a form of scent-marking communication in hamsters (35), and self-grooming.

**Table 1:** Behavior scored during the social interaction test.

Category	Behavior	Description
General activity	Active vertical	Time spent in vertical activity (e.g., rearing).
	Active horizontal locomotor	Time spent in horizontal locomotor activity.
Self-directed behavior	Self-grooming	Time spent grooming the body using forelimbs and mouth.
Non-social exploratory/digging	Bury	Time spent pushing or covering objects/bedding.
	Dig	Time spent actively displacing bedding using forelimbs.
Social investigation	Sniff head	Investigation of the conspecific’s head region.
	Sniff body	Investigation of the conspecific’s body (excluding anogenital region).
	Sniff anogenital	Investigation of the anogenital region of the conspecific.
Affiliative/social interaction	Greet	Initial approach and investigation of a conspecific.
	Allogroom	Grooming directed toward the conspecific.
	Social play	Play behaviors including tumbling, pinning, rolling, and nuzzling.
Scent marking	Flank marking	Rubbing of the flank gland against surfaces to deposit scent marks.
Agonistic behavior	Aggression	Offensive behaviors directed toward the conspecific (e.g., attack, chase).
	Submission	Defensive or submissive responses to aggression (e.g., freezing, retreat, supine posture).
	Total aggressive Score	(Aggression – Submissive behaviors)/(Aggression + Submissive behaviors)

Syrian hamsters exhibit robust social play behavior during the juvenile and early pubertal period, characterized by behaviors such as tumbling, rolling, pinning, and nuzzling (11). The frequency of play behavior increases across development, peaking around PND35 and declining thereafter (9). Accordingly, behavioral testing was conducted at PND35 to capture peak levels of social play and interaction.

#### 2.2.2 Social odor recognition test

The goal of this experiment was to assess the effects of genotype on social recognition and retention of social odor memory.

On PND35, subjects were placed in a clean testing cage (23 × 43 × 20 cm) containing a glass microscope slide (25 × 75 × 1 mm) affixed to the cage wall approximately 2 cm above the floor. During the sample phase, subjects were allowed to investigate two different slides scented with flank gland odor collected from adult conspecifics for 3 min. Female hamsters used as scent donors were in the diestrus phase the day of collection. Odors were obtained within 15 min prior to testing by gently restraining donor animals and rubbing the slide across the flank gland region 20 times. Following the sample phase, subjects were returned to their home cage.

After a 20-min intertrial interval, subjects were tested in the 20-min test phase. Animals were placed back into the same testing cage and presented with two slides: one scented with the odor of a previously encountered conspecific (*familiar odor*) and one scented with the odor of a novel conspecific (*novel odor*). During the test phase, the familiar and novel odor slides were presented on opposite sides of the testing cage. To avoid potential side biases, the left/right position of the familiar and novel odors was counterbalanced across animals. This test phase lasted 3 min.

To assess longer-term retention of social odor memory, the same subjects were tested 24 h later (PND36) in a 24-h test phase. The procedure was identical to the 20-min test phase: the familiar odor was derived from a conspecific encountered during the sample phase on the previous day, and the novel odor was obtained from a previously unencountered conspecific. This test phase also lasted 3 min.

Behavioral measures included time spent sniffing each slide (social investigation), as well as locomotor activity and self-grooming. Social investigation was defined as active sniffing directed toward the slide at a distance of ≤2 mm, including direct contact. This criterion has been widely used to quantify social investigation in social recognition tests (36–38), as it reliably reflects olfactory-based investigation in the confined space of a home-cage-like arena. Because hamsters naturally investigate novel stimuli more than familiar ones (36, 38), duration of stimulus investigation was used to determine whether they recognized the familiar odor during the test. Hence, an investigation ratio (IR) was calculated as IR = N/(N+F), where N is the time spent investigating the novel odor (in sample phases, N represents the slide with the odor that will be replaced) and F is the time spent investigating the familiar odor. Sample phase typically has an IR of roughly 0.5, which is equivalent to the chance level. Significant increases in IR from the average IR of the sample to the IR of the test phase demonstrated familiar odor recognition.

#### 2.2.3 Social avoidance test

Social avoidance testing (5 min) was conducted on PND36, 24 h after defeat training (PND35), as previously described (39). Briefly, defeat training consisted of a 15-min exposure to the home cage of a same-sex resident aggressor (RA). During training, a holding box (used later during testing) was placed in the cage to habituate subjects to the apparatus, and a clear cage top was used to prevent escape. RAs reliably initiated attacks within approximately 30s of the subject’s introduction into the home cage. Following defeat training, subjects were returned to the colony room and left undisturbed until testing the following day.

On the test day (PND36), subjects were placed in a novel cage containing an unfamiliar RA confined within a perforated holding box positioned on one side of the cage. This enclosure allowed visual, olfactory, and auditory contact while preventing physical interaction. Behavior was recorded and later analyzed by observers blind to experimental condition. Social avoidance was assessed by measuring time spent in the half of the cage containing the RA (“near”) versus the opposite half (“far”), with time spent in the “far” zone operationally defined as social avoidance (39, 40). Additional behaviors were quantified, including time spent actively investigating the RA (sniffing through the holes of the holding box), time spent on the holding box without investigation, general locomotor activity, frequency of flank marking, and frequency of stretched approaches (a risk assessment behavior in which the animal extends its body toward the RA while maintaining distance (41–43)). All behavioral measures are described in **Table 2** and were used to assess motivational and affective responses within the social context.

**Table 2:** Behavior scored during the social avoidance test.

Behavior	Description
Near box	Time spent in the half of the cage containing the holding box with the caged resident aggressor (RA).
Sniffing RA	Direct sniffing at the holes of the holding box containing the RA.
Sitting on box	Sitting on top of the holding box without direct investigation of the RA, typically accompanied by sniffing the air or exploration of the cage lid.
Total proximity to RA time	Composite measure of time spent in close proximity to the resident aggressor, calculated as the sum of time spent near the holding box, on the holding box, and directly investigating the RA.
Far from box	Time spent in the half of the cage opposite the holding box containing the RA (operationally defined as *social avoidance*).
Stretched approach	Risk assessment behavior in which the hamster stretches its body toward the holding box containing the RA while keeping the ventral surface close to the floor. The hind feet remain stationary while the head and forelimbs extend forward. This behavior is typically followed by a withdrawal (“step back”) of the forelimbs. Each complete stretch was counted as one stretched approach.
Digging	Rapid forelimb movements displacing bedding material.
Flank marking	The hamster rubs its flank gland region against a surface (e.g., cage wall, floor, or holding box), typically accompanied by a lateral body movement, resulting in deposition of scent marks. Each discrete marking event was counted as one flank mark.
Self-groom	Cleaning movements directed toward the face and/or body using the forelimbs. Both complete and interrupted grooming bouts were included.

### 2.3 Behavioral analysis

All behavioral sessions were video-recorded from above through a clear polycarbonate lid and scored offline using Observer XT software (Noldus Information Technology, Leesburg, VA). Scoring was performed by two observers blind to genotype. Inter-rater reliability exceeded 90% agreement across all measured behaviors. Some behaviors (e.g., digging and flank marking in the social avoidance test) were recorded but occurred too infrequently for statistical analysis and were therefore not included in the results.

### 2.4 Statistical analysis

All statistical analyses were conducted using R and Prism 9 (GraphPad). Behavioral data were examined for normality and homogeneity of variance using Shapiro–Wilk and Levene’s tests, respectively. When assumptions were met, data were analyzed using two-way or mixed-design ANOVAs, with genotype (WT, Het, KO) and sex as between-subject factors. Behavioral paradigm-specific within-subject factors (e.g., phase or odor) were included where appropriate. Significant effects were followed by *post-hoc* comparisons (Tukey’s HSD, Šídák, or Bonferroni adjustment, depending on the analysis). When assumptions of parametric analyses were violated or missing values were present, linear mixed-effects models (REML) were used. Potential outliers were identified using the ROUT (robust regression and outlier removal) method implemented in GraphPad Prism (Q = 1%). Observations identified as outliers were excluded prior to statistical analysis. No outliers were identified in the social interaction or social odor recognition tests. In the social avoidance test, two WT females and one Het male were identified as outliers and excluded from the analyses.

For the social odor recognition test, multiple complementary analyses were conducted to assess recognition memory and behavioral engagement. Investigation ratios (IR = novel investigation time/(novel + familiar investigation time)) were analyzed using mixed-design ANOVAs with phase (sample vs. test) as the within-subject factor and genotype and sex as between-subject factors. Separate analyses were conducted for the 20-min and 24-h retention tests. Investigation times for familiar versus novel odors during the test phase were analyzed using mixed-design ANOVAs with odor as the within-subject factor. Finally, total investigation time was analyzed using mixed-design ANOVAs with phase as the within-subject factor to determine whether differences in recognition performance could be explained by changes in overall exploratory engagement.

For all behavioral results, effect sizes are reported as partial eta squared (ηp^2^), with values of 0.01, 0.06, and 0.14 considered small, medium, and large effects, respectively, based on conventional criteria (44). Data are presented as mean ± SEM and statistical significance was set at p < 0.05.

## 3 Results

### 3.1 V1aR deletion increases social investigation of conspecifics

**Figure 2:**
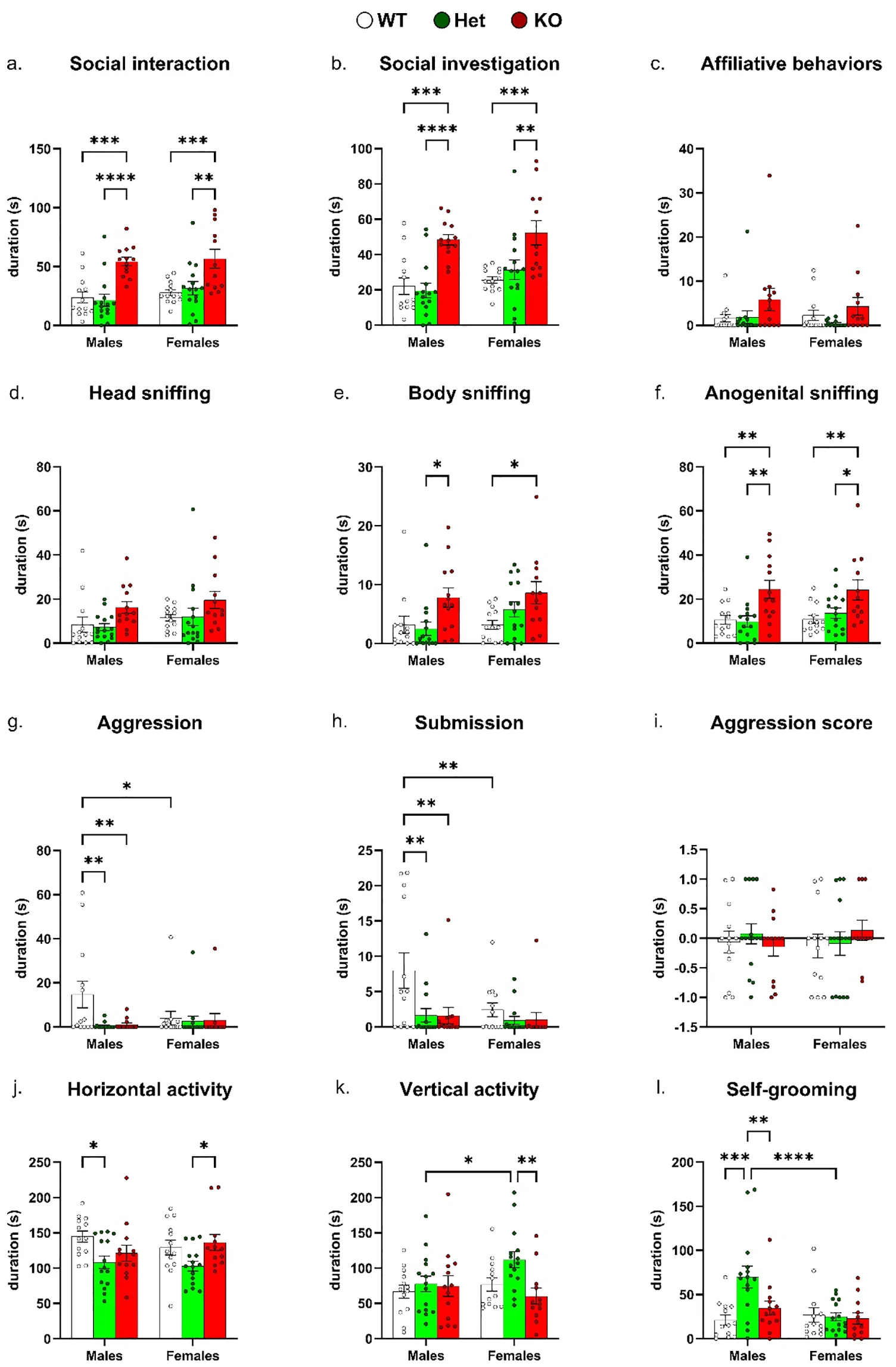
Effects of V1aR knockout on social interaction, investigation, and related behaviors. **(A–C)** Total social interaction, social investigation, and affiliative behaviors. **(D–F)** Specific components of social investigation, including head, body, and anogenital sniffing. **(G–I)** Aggression, submission, and overall aggression score (aggressive minus submissive behaviors over the sum of the two). **(J–L)** Nonsocial behaviors, including locomotor activity, vertical activity, and self-grooming. Data are shown separately for males and females across genotypes (WT, Het, KO). Bars represent mean ± SEM with individual data points overlaid. Significant differences between groups are indicated by asterisks (*p < 0.05, **p < 0.01, ***p < 0.001, ****p < 0.0001). WT males (n = 13), WT females (n = 13), Het males (n = 15), Het females (n = 15), KO males (n = 13), KO females (n = 12).

A two-way ANOVA revealed significant effects of genotype on multiple social behaviors. Total social interaction was strongly affected by genotype (F(2,75) = 20.52, ηp^2^ = 0.35, p < 0.001; **Figure 2A**), as was social investigation (F(2,75) = 19.67, ηp^2^ = 0.34, p < 0.001; **Figure 2B**). In addition, affiliative behavior differed across genotypes (F(2,75) = 3.79, ηp^2^ = 0.09, p = 0.026; **Figure 2C**). More specific measures of social investigation, including sniffing of the head (F(2,75) = 4.65, ηp^2^ = 0.11, p = 0.012; **Figure 2D**), body (F(2,75) = 7.10, ηp^2^ = 0.16, p = 0.002; **Figure 2E**), and anogenital region (F(2,75) = 12.20, ηp^2^ = 0.25, p < 0.001; **Figure 2F**), were also significantly influenced by genotype. *Post-hoc* analyses revealed that both male and female KO hamsters exhibited higher total social interaction compared to WT (p < 0.01) and Het controls (p < 0.001 in males; p < 0.01 in females). This effect was primarily driven by increased social investigation, which showed the same pattern across groups. Within the social investigation domain, KO males spent more time sniffing the body of conspecifics compared to Hets (p < 0.05), whereas KO females showed increased body sniffing relative to WT controls (p < 0.05). The most pronounced effect was observed for anogenital investigation: KO males spent more time sniffing the anogenital region compared to both WT (p < 0.01) and Het (p < 0.01) animals, and KO females exhibited a similar pattern, spending more time sniffing than WT (p < 0.01) and Het (p < 0.05) controls.

Genotype significantly affected low-frequency aggressive (F(2,75) = 3.97, ηp^2^ = 0.10, p = 0.02; **Figure 2G**) and submissive behaviors (F(2,75) = 5.85, ηp^2^ = 0.14, p = 0.004; **Figure 2H**), although the overall incidence of these behaviors was limited. A main effect of sex was also observed for submissive behavior (F(1,75) = 4.49, ηp^2^ = 0.06, p = 0.03). Tukey’s HSD *post-hoc* analysis revealed that WT males exhibited higher levels of aggression compared to Het and KO males (p < 0.01), as well as WT females (p < 0.05). A similar pattern was observed for submissive behavior, with WT males displaying higher levels than Het males, KO males, and WT females (all p < 0.05). However, the overall aggressive score (*aggressive minus submissive behaviors over the sum of the two*) did not differ across genotypes or sexes (all p > 0.05; **Figure 2I**), suggesting that these effects were subtle and did not reflect robust changes in overall agonistic behavior.

Genotype also influenced certain nonsocial behaviors, including locomotor activity (F(2,75) = 6.20, ηp^2^ = 0.14, p = 0.003; **Figure 1J**) and vertical activity (F(2,75) = 3.47, ηp^2^ = 0.09, p = 0.03; **Figure 2K**). Self-grooming behavior was significantly affected by genotype (F(2,75) = 4.84, ηp^2^ = 0.11, p = 0.01; **Figure 2L**). *Post-hoc* analyses indicated that WT males exhibited greater locomotor activity compared to Het males (p < 0.05), whereas KO females showed higher locomotor activity than Het females (p < 0.05). For vertical activity, Het females exhibited higher levels than both KO females (p < 0.01) and Het males (p < 0.05).

A main effect of sex was also observed for self-grooming (F(1,75) = 6.55, ηp^2^ = 0.08, p = 0.01; **Figure 2L**). In addition, a significant genotype × sex interaction was detected (F(2,75) = 5.25, ηp^2^ = 0.12, p = 0.007), indicating that genotype effects on grooming behavior differed between males and females. *Post-hoc* analysis showed that Het males exhibited higher self-grooming behavior compared to WT (p < 0.001) and KO (p < 0.01) males, as well as Het females (p < 0.001). Interestingly, Het males exhibited a distinct behavioral profile characterized by reduced locomotion alongside increased vertical activity and self-grooming, suggesting a shift from active exploration toward heightened vigilance or arousal-related behaviors.

No significant effects of genotype or sex were observed for digging, burying, play behavior, general activity, non-social activity, or flank-marking frequency (all p > 0.05).

### 3.2 Heterozygous hamsters show impaired social odor recognition 20 minutes after sample exposure

**Figure 3:**
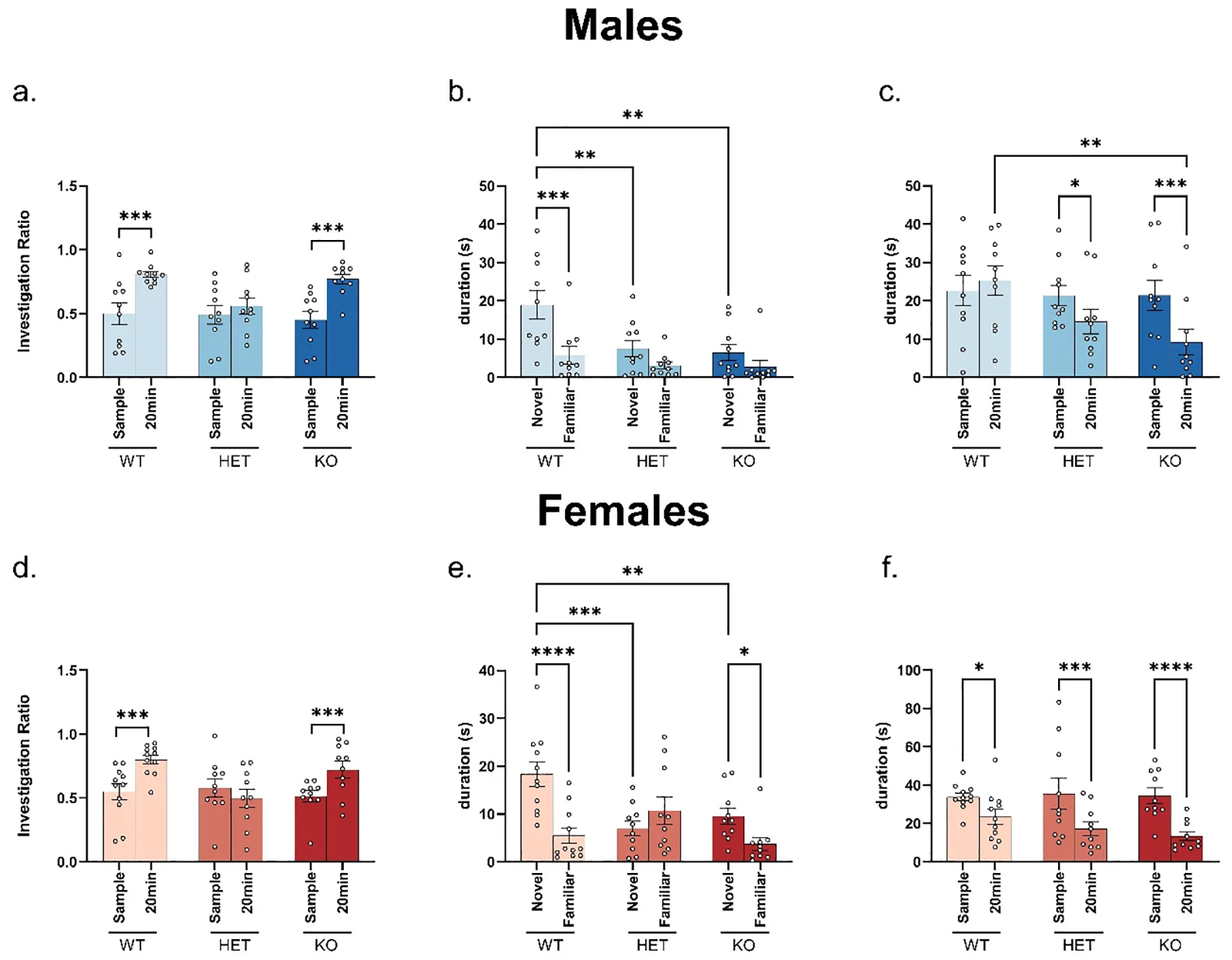
Effects of V1aR knockout on short-term social odor recognition (20-min test). **(A, D)** Investigation ratio (IR) during the sample and test phases at 20 min. **(B, E)** Time spent investigating familiar versus novel odors during the test phase. **(C, F)** Total investigation time across phases (sample and test). Data are shown separately for males (*upper panel*) and females (*bottom panel*) across genotypes (WT, Het, KO). Bars represent mean ± SEM with individual data points overlaid. Significant differences are indicated by asterisks (*p < 0.05, **p < 0.01, ***p < 0.001). WT males (n = 10), WT females (n = 11), Het males (n = 10), Het females (n = 10), KO males (n = 10), KO females (n = 10).

A mixed-design ANOVA revealed a significant main effect of genotype (F(2,55) = 6.12, ηp^2^ = 0.18, p = 0.004; **Figures 3A, D**) and a significant main effect of phase (sample vs test; F(1,55) = 21.55, ηp^2^ = 0.28, p < 0.001), indicating overall differences in investigation ratio across genotypes and between the initial sample exposure and the subsequent test phase. Importantly, a significant genotype × phase interaction (F(2,55) = 5.77, ηp^2^ = 0.17, p = 0.005) indicated that social odor recognition differed across genotypes. No main effect of sex (F(1,55) = 0.18, p = 0.67) and no significant interactions involving sex (all p > 0.18) were observed (**Figures 3A, D**). *Post-hoc* comparisons revealed that WT (p < 0.001) and KO (p < 0.001) animals of both sexes showed significant differences between the sample and test phases, indicating intact social odor recognition. In contrast, Het animals did not differ between phases (p = 0.92), suggesting impaired recognition memory.

To directly assess social odor discrimination, time spent investigating familiar versus novel odors during the sample and test phase was analyzed using a mixed-design ANOVA (data not shown). During the sample phase, no significant differences in investigation time were observed between the two odors (F(1,55) = 1.04, ηp^2^ = 0.02, p = 0.313), indicating no inherent odor preference. There were also no significant interactions between odor and genotype or sex (all p > 0.49). A main effect of sex was observed (F(1,55) = 12.75, ηp^2^ = 0.19, p < 0.001), reflecting overall differences in investigation time, but this did not interact with odor, suggesting that baseline odor preference was not influenced by sex. During the test phase, a two-way mixed ANOVA revealed a significant main effect of odor (F(1,55) = 29.57, ηp^2^ = 0.35, p < 0.001; **Figures 3B, E**), indicating increased investigation of the novel stimulus compared to the familiar one. A significant genotype × odor interaction was also observed (F(2,55) = 11.55, ηp^2^ = 0.30, p < 0.001), suggesting that social recognition differed across genotypes. *Post-hoc* analyses in males showed that WT animals exhibited robust discrimination between the novel and familiar odors (p < 0.001), whereas Het (p = 0.15) and KO (p = 0.23) animals showed no preference, indicating impaired social recognition. In addition, WT hamsters spent more time investigating the novel odor than both Het and KO animals (p < 0.01). In females, *post-hoc* analyses indicated that WT (p < 0.001) and KO (p < 0.05) animals showed significant discrimination between the two odors, whereas Het animals did not (p = 0.11). WT females also spent more time investigating the novel odor than both Het (p < 0.001) and KO (p < 0.01) animals.

Total investigation time across phases was analyzed using a mixed-design ANOVA (**Figures 3C, F**). No significant main effect of genotype was observed (F(2,55) = 1.90, p = 0.16), indicating that overall investigation levels did not differ across groups. However, a significant main effect of sex (F(1,55) = 6.47, ηp^2^ = 0.10, p = 0.014) and a robust effect of phase (F(1,55) = 47.29, ηp^2^ = 0.46, p < 0.001) were detected. Importantly, a significant genotype × phase interaction (F(2,55) = 5.15, ηp^2^ = 0.16, p = 0.009) indicated that changes in total investigation across phases differed between genotypes. A significant sex × phase interaction was also observed (F(1,55) = 12.50, ηp^2^ = 0.18, p < 0.001), suggesting that males and females differed in their pattern of investigation across phases. No genotype × sex or three-way interactions were detected (all p > 0.80). Follow-up comparisons revealed that both Het (p < 0.05) and KO (p < 0.001) males showed significant reductions in total investigation time from the sample to the test phase, whereas WT animals did not (p = 0.39). In addition, KO males showed lower total investigation than WT animals at the 20-min timepoint (p < 0.01). In females, all three genotypes showed a reduction of total time spent investigating during the test in comparison to sample phase (WT, p < 0.05; Het, p < 0.001; KO, p < 0.001). These findings indicate that, although baseline investigation levels were comparable across genotypes, Het and KO animals exhibited a marked decrease in overall engagement during the test phase relative to WT controls.

Together, these findings indicate a selective impairment in short-term social recognition in heterozygous animals, despite preserved baseline investigation levels.

### 3.3 Social odor recognition is not retained at 24 h across genotypes

**Figure 4:**
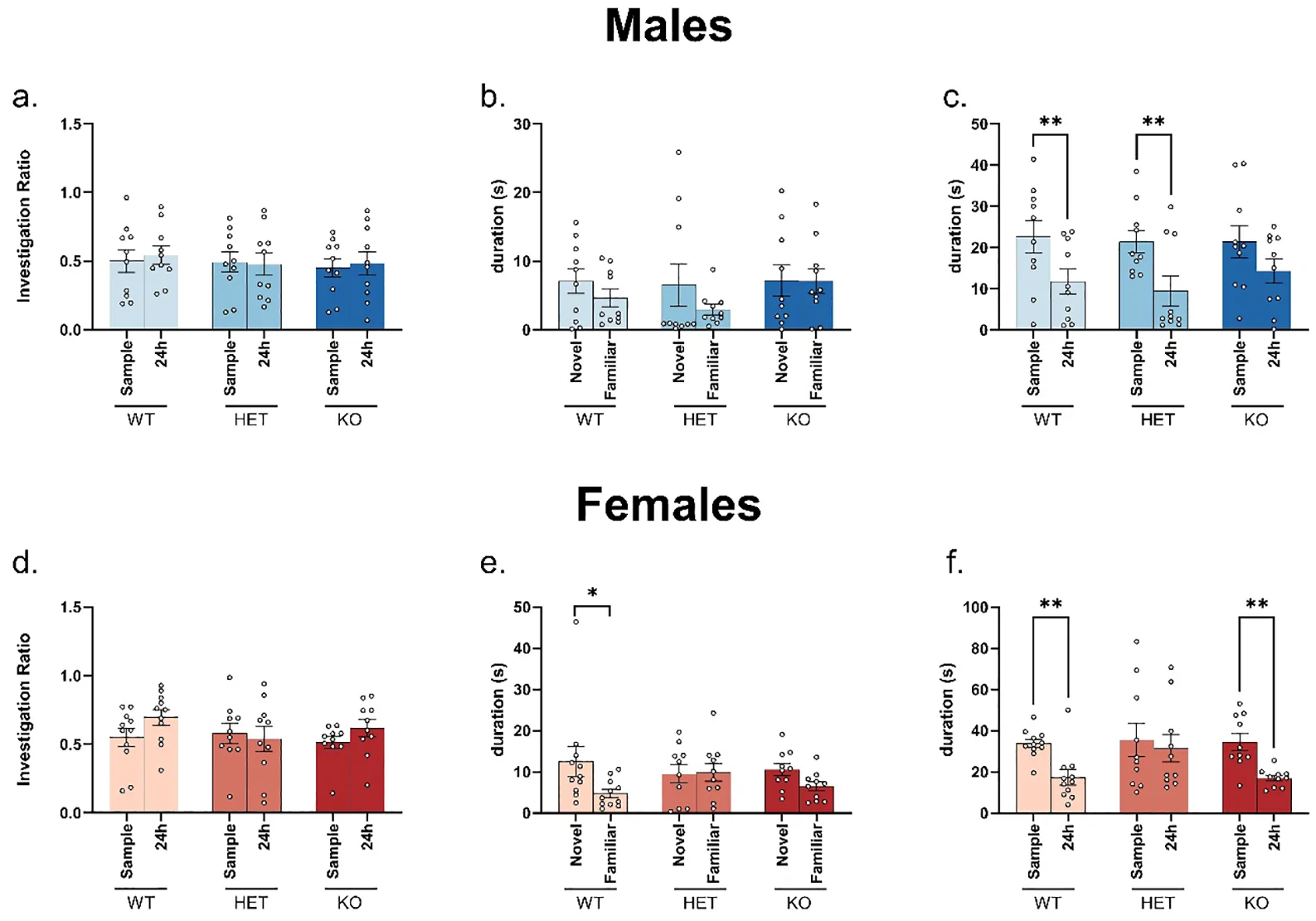
Effects of V1aR knockout on long-term social odor recognition (24-h test). **(A, D)** Investigation ratio (IR) during the sample and test phases at 24 h. **(B, E)** Time spent investigating familiar versus novel odors during the test phase. **(C, F)** Total investigation time across phases (sample and test). Data are shown separately for males (*upper panel*) and females (*bottom panel*) across genotypes (WT, Het, KO). Bars represent mean ± SEM with individual data points overlaid. Significant differences are indicated by asterisks (*p < 0.05, **p < 0.01, ***p < 0.001). WT males (n = 10), WT females (n = 11), Het males (n = 10), Het females (n = 10), KO males (n = 10), KO females (n = 10).

To assess long-term retention of social odor memory, IRs were analyzed 24 h after the sample phase using a mixed-design ANOVA. No significant main effect of genotype (F(2,55) = 0.69, p = 0.51) or phase (F(1,55) = 2.55, p = 0.12; **Figures 4A, D**) was observed. Importantly, no genotype × phase interaction was detected (F(2,55) = 1.39, p = 0.26), indicating that none of the groups exhibited differential changes in investigation across phases. A modest main effect of sex was observed (F(1,55) = 4.68, ηp^2^ = 0.08, p = 0.035), whereas no other interactions involving sex were significant (all p > 0.29). Together, these findings indicate that social odor recognition was not retained at the 24-h time point across genotypes.

At the 24-h test, a three-way mixed ANOVA revealed a modest but significant main effect of odor (F(1,55) = 6.11, ηp^2^ = 0.10, p = 0.017), indicating a general preference for the novel odor (**Figures 4B, E**). However, in contrast to the 20-min test, no significant genotype × odor interaction was observed (F(2,55) = 0.88, p = 0.422), suggesting that genotype did not modulate long-term social recognition. A main effect of sex was detected (F(1,55) = 6.22, ηp^2^ = 0.10, p = 0.016), reflecting overall differences in investigation time, but this did not interact with odor. No main effect of genotype was found (F(2,55) = 0.10, p = 0.908). *Post-hoc* comparisons indicated that only female WT animals retained a significant preference for the novel odor (p = 0.023), whereas Het and KO animals did not (p > 0.05), although these differences should be interpreted cautiously in the absence of a significant interaction.

Total investigation time across phases at the 24-h time point was analyzed using a mixed-design ANOVA (**Figures 4C, F**). No significant main effect of genotype was observed (F(2,55) = 0.01, ηp^2^ = 0.0004, p = 0.99), and no genotype × phase interaction was detected (F(2,55) = 0.08, ηp^2^ = 0.003, p = 0.92), indicating that overall investigation levels and their changes across phases did not differ between genotypes. A significant main effect of sex (F(1,55) = 14.60, ηp^2^ = 0.21, p < 0.001) and a robust effect of phase (F(1,55) = 52.15, ηp^2^ = 0.49, p < 0.001) were observed. A trend toward a sex × phase interaction was also detected (F(1,55) = 3.44, ηp^2^ = 0.06, p = 0.069), whereas no other interactions were significant (all p > 0.77). Overall, animals showed reduced total investigation during the 24-h test phase compared with the sample phase, regardless of genotype. Exploratory Šídák-corrected *post-hoc* comparisons showed significant reductions in WT and Het males (both p < 0.01), with a similar trend in KO males (p = 0.057). In females, WT and KO animals also showed reduced total investigation (both p < 0.01), whereas Het females did not. These findings should be interpreted cautiously, however, given the absence of a significant genotype × phase interaction.

These findings differ from previous studies in Syrian hamsters reporting robust social recognition at 24 h but not 48 h using habituation-based paradigms (31). The present study employed a choice-based task, which may engage partially distinct cognitive and motivational processes, potentially accounting for the reduced long-term retention observed here.

### 3.4 Male heterozygous hamsters exhibit increased social avoidance

**Figure 5:**
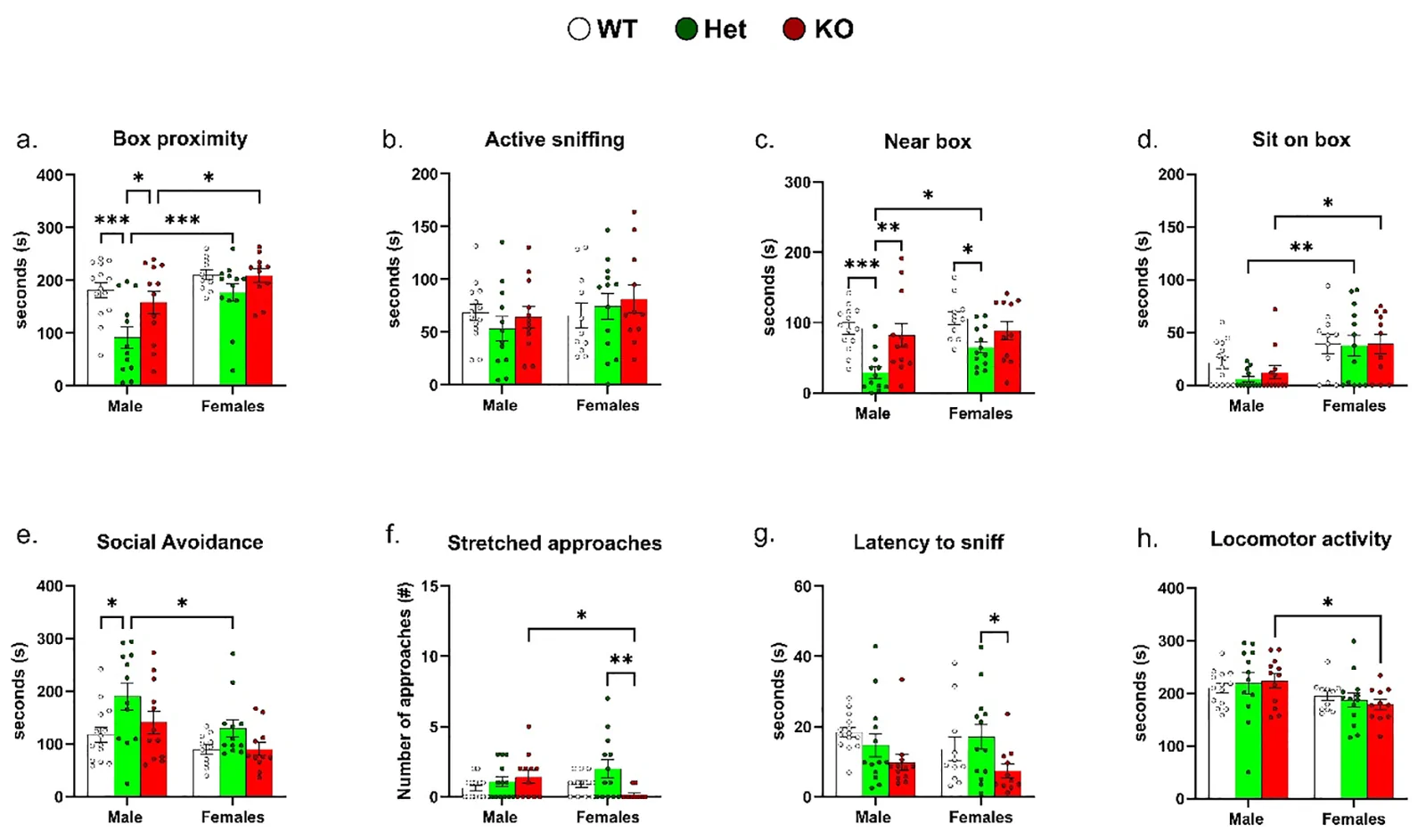
Effects of V1aR knockout on social avoidance behavior following social defeat. **(A)** Total time spent in proximity to the resident aggressor (RA), defined as the sum of time spent near the holding box, sitting on the box, and actively investigating the RA. **(B)** Time spent actively investigating the RA. **(C)** Time spent near the holding box. **(D)** Time spent on the holding box without investigation. **(E)** Time spent far from the holding box (operationally defined as social avoidance). **(F)** Number of approaches toward the holding box. **(G)** Latency to the first investigation of the RA. Data are shown separately for males and females across genotypes (WT, Het, KO). Bars represent mean ± SEM with individual data points overlaid. Significant differences are indicated by asterisks (*p < 0.05, **p < 0.01, ***p < 0.001). WT males (n = 14), WT females (n = 11), Het males (n = 12), Het females (n = 13), KO males (n = 12), KO females (n = 11).

A two-way ANOVA revealed significant effects of genotype on multiple aspects of social avoidance, with several sex-specific effects. Total time spent in proximity to the holding box (near + sitting on + actively sniffing the resident aggressor) was affected by both genotype (F(2,67) = 7.85, ηp^2^ = 0.19, p < 0.001; **Figure 5A**) and sex (F(1,67) = 16.14, ηp^2^ = 0.19, p < 0.001). *Post-hoc* comparisons indicated that Het males spent less time near the holding box compared to male WT (p < 0.001), male KO (p < 0.05), and female Hets (p < 0.001). In addition, there was a sex difference in the KO groups, with females spending more time near the box than males (p < 0.05).This effect was driven by time spent near the holding box rather than active investigation (all p > 0.05; **Figure 5B**). Specifically, time spent near the box was significantly affected by genotype (F(2,67) = 12.44, ηp^2^ = 0.27, p < 0.001; **Figure 5C**) and sex (F(1,67) = 4.35, ηp^2^ = 0.06, p = 0.041), with no genotype × sex interaction. *Post-hoc* analyses revealed that Het males spent less time near the box compared to WT males (p < 0.001) and KOs (p < 0.01), and that Het females spent less time near the box than WT females (p < 0.05). In addition, males spent less time near the box than females within the Het group (p < 0.05). Sex also significantly affected time spent on the holding box without investigating the resident aggressor (F(1,67) = 17.40, ηp^2^ = 0.21, p < 0.001; **Figure 5D**), with both Het and KO males spending less time on the box than their female counterparts (Het: p < 0.01; KO: p < 0.05).

Social avoidance (time spent in the half of the cage opposite the holding box) was significantly affected by genotype (F(2,67) = 5.59, ηp^2^ = 0.14, p = 0.005; **Figure 5E**) and sex (F(1,67) = 10.49, ηp^2^ = 0.13, p = 0.002). *Post-hoc* comparisons indicated that Het males exhibited increased avoidance compared to male WT (p < 0.05) and female Het hamsters (p < 0.05).

The number of approaches to the holding box was not affected by genotype or sex, but a significant genotype × sex interaction was detected (F(2,67) = 4.05, ηp^2^ = 0.11, p = 0.021; **Figure 5F**). *Post-hoc* analysis revealed that KO females made fewer approaches than did Het females (p < 0.01) and KO males (p < 0.05).

Latency to the first sniff of the resident aggressor was affected by genotype (F(2,67) = 3.93, ηp^2^ = 0.11, p = 0.024; **Figure 5G**), with no effect of sex or interaction. *Post-hoc* comparisons indicated reduced latency in KO animals, driven primarily by KO females compared to Het animals (p < 0.05), with a trend toward reduced latency in KO males compared to WT males (p = 0.06).

Finally, total locomotor activity during the social avoidance test was quantified to determine whether differences in avoidance behavior could be explained by generalized changes in activity (**Supplementary Figure S1**). A significant effect of sex was observed (F(1,70) = 5.442, ηp^2^ = 0.07, p < 0.05; **Figure 5H**), whereas no effect of genotype or genotype × sex interaction was detected (all p > 0.05). *Post-hoc* analyses indicated lower locomotor activity in KO females compared to KO males (p < 0.05). These findings suggest that the genotype-dependent differences in social avoidance are unlikely to reflect generalized alterations in locomotor activity.

No other behavioral measures were significantly affected.

## 4 Discussion

Our findings indicate that V1aR disruption produces domain-specific and nonlinear effects on juvenile hamster social behavior. Across assays, heterozygous animals often showed the clearest phenotype, with altered social interaction, impaired short-term social recognition, and increased social avoidance, whereas knockout animals showed a more mixed pattern. This overall profile fits with the broader view that vasopressin signaling does not regulate “sociality” uniformly, but instead modulates partially dissociable components of social cognition, social motivation, and social valence within the social behavior neural network (17, 45). Importantly, these findings were observed during the juvenile period, a developmental stage characterized by heightened social engagement and ongoing maturation of neural circuits underlying social behavior. Social play and social investigation during this period are thought to contribute to the refinement of adult social competence, including social communication, stress coping, and context-appropriate behavioral responses. Studying V1aR function at this stage therefore provides insight not only into the regulation of social behavior per se, but also into mechanisms that may shape the developmental trajectory of social circuits.

Importantly, the largest effect sizes were consistently observed for measures of social investigation and social odor recognition, indicating that V1aR genotype accounted for a substantial proportion of the variance in these behaviors. By contrast, effect sizes for aggressive, submissive, and several nonsocial behaviors were generally smaller, consistent with our interpretation that V1aR disruption primarily influences social information processing rather than broadly altering all behavioral domains. The observation that V1aR disruption alters multiple domains of social behavior in juveniles suggests that vasopressin signaling may play a critical role in organizing social behavior early in life with potential long-term consequences.

The social interaction data suggest that V1aR contributes importantly to social investigation, particularly toward socially informative body regions. This is consistent with a large literature linking vasopressin signaling to social communication, stimulus salience, and aggression-related behavior in hamsters and other rodents (17, 46, 47). In our study, knockout animals showed elevated social investigation, especially anogenital investigation, whereas heterozygous males displayed a distinct pattern of reduced locomotor activity, elevated vertical activity, and increased self-grooming. Rather than reflecting a simple increase or decrease in sociability, this pattern suggests that partial disruption of V1aR signaling may alter the balance between social approach, environmental scanning, and arousal-related behavior. The genotype × sex interaction observed for self-grooming may reflect sex-dependent recruitment of AVP-sensitive arousal and stress-regulation circuits rather than a direct effect on grooming motor output alone. Self-grooming is commonly expressed during heightened arousal, conflict, or stress, and AVP signaling influences these states through distributed hypothalamic, septal, amygdalar, and brainstem pathways (48, 49). Because extrahypothalamic AVP projections arising from the bed nucleus of the stria terminalis and medial amygdala can be sexually differentiated and steroid sensitive (50), partial V1aR disruption may alter the balance between social engagement and arousal-related coping more prominently in juvenile males. The combination of reduced locomotion and increased vertical activity and grooming in Het males is therefore consistent with altered behavioral-state regulation, although the present study cannot identify the specific circuit responsible. The relatively large effect sizes observed for total social investigation and anogenital investigation further support the conclusion that V1aR signaling plays a prominent role in the processing of socially relevant olfactory cues during juvenile social interactions. This interpretation is also consistent with the broader vasopressin literature, which has repeatedly shown that V1aR signaling can affect both social and nonsocial responses depending on context and circuit locus (17, 19).

Although genotype effects were also detected for aggressive and submissive behaviors, these behaviors occurred at relatively low frequencies during the juvenile interaction test and did not translate into differences in the overall aggression score. Therefore, these findings should be interpreted cautiously and likely reflect subtle shifts in social responsiveness rather than robust alterations in agonistic behavior. Juvenile Syrian hamsters generally display limited overt aggression under neutral testing conditions (51–53), and the brief interaction paradigm used here was designed primarily to assess social investigation and play-related behaviors rather than territorial aggression. Future studies specifically optimized to assess agonistic behavior may be necessary to clarify whether V1aR disruption meaningfully alters aggression-related responses during development.

Our social recognition results are especially informative because they point to a time-dependent and nonlinear genotype effect. At 20 min, heterozygous hamsters failed to show normal recognition, whereas wildtype animals exhibited robust discrimination and knockout animals showed intact or partially preserved recognition depending on the metric used. This finding both aligns with and departs from prior work. In mice, constitutive V1aR deletion produces profound social recognition deficits, and restoration of V1aR expression in the lateral septum is sufficient to rescue recognition, strongly implicating septal V1aR signaling in social memory processing (19, 23). However, more recent CRISPR-based hamster studies have shown that deleting V1aR can produce paradoxical social phenotypes rather than straightforward deficits, underscoring the importance of species, context, and developmental compensation (32, 45, 54). Our results fit that newer hamster literature better than the classic mouse knockout literature: the strongest deficit emerged in heterozygotes rather than in knockouts, suggesting that partial V1aR disruption may dysregulate social memory circuits in a way that differs from complete receptor loss.

One plausible interpretation is that heterozygous disruption exposes a dosage-sensitive phenotype, whereas full knockout permits compensatory adaptation. This kind of non-monotonic effect is consistent with the growing realization that peptide systems often show nonlinear relationships between receptor expression and behavior, particularly when manipulations are present throughout development (32, 45, 55). In that sense, our data argue against a simple loss-of-function model in which less V1aR necessarily means less recognition. Instead, they suggest that partial receptor reduction may create an intermediate state in which social information processing becomes particularly vulnerable to circuit imbalance. This interpretation is also consistent with our avoidance data, where heterozygous males again showed the strongest phenotype. The magnitude of these effects further supports this interpretation. In particular, the large effect sizes associated with odor discrimination and the genotype × odor interaction indicate that the impairment observed in heterozygous animals represents a robust behavioral phenotype rather than a marginal statistical effect. Together, these findings suggest that partial disruption of V1aR signaling produces biologically meaningful alterations in social information processing.

An additional possibility is that heterozygous animals retain sufficient receptor expression to preserve partially functional vasopressin circuits, but not enough to maintain normal network dynamics. Because V1aRs are distributed across multiple nodes of the social behavior network, including the lateral septum, amygdala, hypothalamus, ventral pallidum, and reward-related midbrain regions, the behavioral consequences of receptor reduction are unlikely to be spatially uniform (17, 23, 49). A partial reduction could move some circuits below the signaling threshold required for normal function while leaving others relatively intact, thereby producing an imbalance across systems regulating social salience, recognition, arousal, and avoidance. For example, reduced septal V1aR signaling could impair the encoding or retrieval of socially relevant olfactory information, whereas preserved or differently compensated signaling in amygdalar or hypothalamic circuits could maintain or even enhance investigation and defensive responsiveness (17, 23).

By contrast, complete receptor loss throughout development may evoke broader homeostatic adaptations, including altered AVP release, changes in oxytocin or V1b receptor signaling, receptor-independent network reorganization, or compensatory adjustments in excitatory and inhibitory transmission (55). Such compensation could reduce the behavioral impact of complete receptor deletion while leaving heterozygous animals in an incompletely compensated state. The CRISPR model used here has been validated as a functional null in knockout animals through the absence of detectable V1aR binding and loss of central and peripheral responses to V1aR activation, arguing against substantial receptor “leakiness” in the KO condition (32). However, receptor abundance and signaling capacity have not been mapped across individual brain regions in heterozygous animals. Therefore, the extent to which gene dosage produces uniform versus circuit-specific reductions in V1aR function remains unknown. Future studies combining regional receptor quantification with circuit-restricted rescue or knockdown approaches will be needed to test this model directly (23, 49).

In contrast to prior work in Syrian hamsters demonstrating robust social recognition at 24 h but not 48 h (31), we did not observe strong evidence of long-term retention in any genotype. One important methodological difference is that previous studies have typically used habituation–dishabituation paradigms and have tested adults, whereas the present study employed a choice-based recognition task in prepubertal animals (46, 56). These paradigms may rely on partially distinct neural and motivational processes, with habituation procedures emphasizing repeated exposure and familiarity encoding, and choice tasks requiring active discrimination between competing stimuli. It is therefore possible that the lack of robust 24-h recognition in the present study reflects task-specific differences rather than an absence of long-term social memory per se. In addition, short- and long-term social recognition are also likely to depend on partially distinct neural and molecular processes. V1aR signaling within the lateral septum appears especially important for the detection and early encoding of socially relevant information (57), whereas longer-term retention may additionally require coordinated activity and protein-dependent plasticity across hippocampal, prefrontal, and amygdalar circuits (58). For example, hippocampal CA2–CA1 pathways contribute prominently to social memory, while protein synthesis in the medial prefrontal cortex, anterior cingulate cortex, and basolateral amygdala appears to be more important for long-term than short-term social recognition (58). Thus, partial V1aR disruption may impair the rapid assignment of salience or encoding of social odors, whereas the limited 24-h retention observed across all genotypes may reflect weaker recruitment of broader consolidation networks under the present juvenile choice-based paradigm. Future studies directly comparing these paradigms within the same animals and across development will be important for clarifying the role of V1aR in long-term social memory.

The total-investigation analyses are also informative because they help interpret the recognition phenotype. At 20 min, heterozygous and knockout animals showed reduced total investigation at test relative to sample, whereas wildtypes did not. This indicates that genotype can affect engagement across repeated exposure, not only discrimination per se. However, this does not fully account for the recognition phenotype, because heterozygotes were impaired in both IR-based and familiar-versus-novel analyses, whereas knockouts still showed evidence of recognition despite reduced total investigation. Thus, altered engagement may contribute to the phenotype, but it cannot fully explain the selective short-term recognition deficit in heterozygotes.

The social avoidance findings further strengthen the interpretation that V1aR regulates social valence and approach-avoidance balance, especially under stressful conditions. Heterozygous males showed the clearest avoidance phenotype, spending less time near the aggressor and more time in the avoidance zone. This fits well with the Syrian hamster defeat literature, which has long shown that prior defeat produces robust conditioned social avoidance and that this paradigm is sensitive to neuroendocrine modulation (39, 40, 59). It also converges with developmental hamster work showing that perturbations in juvenile social experience can increase defeat-induced avoidance later in life (10, 60). In our case, the phenotype is genetic rather than experiential, but both sets of findings point to the idea that social approach after challenge depends on mechanisms that overlap with, but are not identical to, those regulating social investigation and recognition.

Taken together, the three behavioral domains assessed here support a model in which V1aR contributes to social information processing, social engagement, and social coping, but in a manner that is strongly shaped by receptor dosage and behavioral context. Rather than producing a single global “social deficit,” V1aR disruption altered some domains more than others, and, in several cases, heterozygotes were more affected than knockouts. This domain specificity is highly consistent with current frameworks emphasizing that oxytocin and AVP systems act across distributed social circuits, with different nodes contributing to recognition, affiliation, aggression, and stress-related avoidance (17, 49).

Although both sexes were included, sex effects were relatively modest and behavior-specific. Sex differences were observed in certain measures, such as overall investigation and social avoidance-related behaviors, but were not a dominant factor across all assays. Notably, the key genotype-dependent effects on social recognition were not modulated by sex, suggesting that V1aR contributes similarly to social memory processes in males and females at this developmental stage. This is somewhat unexpected given well-established sex differences in AVP systems, including sexually dimorphic AVP projections and receptor expression within key nodes of the social behavior network such as the lateral septum (17, 27, 61, 62). In Syrian hamsters specifically, AVP has been strongly implicated in the regulation of social communication, aggression, and stress-related social behaviors, often in a sex-dependent manner (17, 39). However, accumulating evidence suggests that sex differences in AVP function are highly context- and behavior-specific, particularly during juvenile development (63, 64). One possibility is that more robust sex differences emerge later in development or under adult hormonal conditions, as AVP systems undergo substantial maturation and steroid-dependent modulation (65, 66).

The developmental trajectory of AVP signaling may also differ across species. In rodents, sexually differentiated AVP projections arising from the BNST and medial amygdala develop under the influence of gonadal steroids and become increasingly pronounced during pubertal maturation, although the timing, receptor distribution, and behavioral consequences of these changes are not identical across mice, rats, and Syrian hamsters (17, 27, 61, 65, 66). Juvenile animals may therefore exhibit less pronounced or less consistent sex differences than adults, even when specific behaviors already show sex-dependent sensitivity to V1aR disruption. In mice, constitutive V1aR deletion produces relatively robust deficits in social recognition that can be rescued by restoring V1aR expression within the lateral septum (22). By contrast, CRISPR-mediated V1aR deletion in Syrian hamsters has produced more complex and behavior-specific phenotypes, including nonlinear effects that differ between heterozygous and knockout animals (32, 49, 54). These differences likely reflect a combination of species-specific receptor distribution, circuit organization, and developmental compensation (17, 49). Consequently, findings from mouse knockout models should not necessarily be expected to translate directly to juvenile Syrian hamsters. Alternatively, testing animals prior to the onset of stable estrous cyclicity may have reduced variability and minimized sex-dependent effects. However, our results highlight the importance of including both sexes while also suggesting that V1aR-dependent regulation of juvenile social behavior may be less sexually differentiated than in adults.

Several limitations should be acknowledged. First, because this is a constitutive, germline manipulation, developmental compensation remains a plausible explanation for why knockout animals did not uniformly show stronger phenotypes than did heterozygotes. Second, while both sexes were included, most of the strongest sex-linked effects emerged in overall investigation or avoidance-related measures rather than in recognition itself, so sex-specific circuit mechanisms remain to be clarified. Third, home-cage social rank was not prospectively assessed. Although animals were tested in a neutral arena with unfamiliar, weight-matched conspecifics rather than with their cage mates, prior social experience within the home cage may nevertheless have contributed to individual variability in social behavior. Because social dominance relationships were not systematically evaluated, we cannot exclude a potential influence of home-cage social status on some behavioral measures. Future studies incorporating longitudinal observations of home-cage interactions or validated dominance assays will be important to determine whether social rank interacts with V1aR genotype. Fourth, the social recognition task revealed limited evidence of 24-h retention overall, which constrains conclusions about long-term memory. Future work using region-specific or temporally restricted V1aR manipulations, especially within the lateral septum and related olfactory-social circuits, will be important for resolving whether the heterozygote phenotype reflects altered encoding, altered salience assignment, or altered behavioral expression of social memory.

In summary, our results extend recent CRISPR findings in hamsters by showing that V1aR disruption affects multiple facets of social behavior within the same experimental framework. The most prominent phenotype emerged in heterozygous animals, which showed reduced social engagement, impaired short-term social recognition, and increased social avoidance. A nonlinear, context-dependent role for V1aR in the regulation of social behavior emerged and partial disruption of AVP signaling may be as behaviorally meaningful as complete receptor loss. Together, these findings emphasize that V1aR function during juvenile development shapes multiple facets of social behavior in a manner that is both nonlinear and only modestly influenced by sex.

## Data Availability

The raw data supporting the conclusions of this article will be made available by the authors, without undue reservation.

## References

[1] Insel TR Fernald RD . How the brain processes social information: Searching for the social brain. Annu Rev Neurosci. (2004) 27:697–722. doi: 10.1146/annurev.neuro.27.070203.144148 15217348

[2] O’Connell LA Hofmann HA . The vertebrate mesolimbic reward system and social behavior network: A comparative synthesis. J Comp Neurol. (2011) 519(18):3599–639. doi: 10.1002/cne.22735 21800319

[3] Arioli M Crespi C Canessa N . Social cognition through the lens of cognitive and clinical neuroscience. BioMed Res Int. (2018) 2018:4283427. doi: 10.1155/2018/4283427 30302338 PMC6158937

[4] Crider A Pillai A . The neurobiological basis for social affiliation in autism spectrum disorder and schizophrenia. Curr Behav Neurosci Rep. (2016) 3:154. doi: 10.1007/S40473-016-0079-0 27695666 PMC5042200

[5] Modi ME Young LJ . The oxytocin system in drug discovery for autism: Animal models and novel therapeutic strategies. Horm Behav. (2011) 61:340. doi: 10.1016/J.YHBEH.2011.12.010 22206823 PMC3483080

[6] Abel KM Drake R Goldstein JM . Sex differences in schizophrenia. Int Rev Psychiatry. (2010) 22(5):417–28. doi: 10.3109/09540261.2010.515205 21047156

[7] Gogos A Ney L Seymour N Van Rheenen T Felmingham K . Sex differences in schizophrenia, bipolar disorder, and post-traumatic stress disorder: Are gonadal hormones the link? Br J Pharmacol. (2019) 176:4119–35. doi: 10.1111/BPH.14584 30658014 PMC6877792

[8] Green T Flash S Reiss AL . Sex differences in psychiatric disorders: what we can learn from sex chromosome aneuploidies. Neuropsychopharmacology. (2019) 44:9. doi: 10.1038/S41386-018-0153-2 30127341 PMC6235860

[9] Cooper MA Grizzell JA Whitten CJ Burghardt GM . Comparing the ontogeny, neurobiology, and function of social play in hamsters and rats. Neurosci Biobehav Rev. (2023) 147:105102. doi: 10.1016/J.NEUBIOREV.2023.105102 36804399 PMC10023430

[10] Kyle SC Burghardt GM Cooper MA . Development of social play in hamsters: Sex differences and their possible functions. Brain Res. (2019) 1712:217–23. doi: 10.1016/J.BRAINRES.2019.02.012 30768930 PMC6461509

[11] Onyekwere DI Ramírez JM . Play fighting versus serious fighting in golden hamsters (Mesocricetus auratus). Bull Psychonomic Soc. (2013) 31:503–6. doi: 10.3758/BF03337336

[12] Pellis SM Pellis VC . Play-fighting in the Syrian golden hamster Mesocricetus auratus waterhouse, and its relationship to serious fighting during postweaning development. Dev Psychobiol. (1988) 21:323–37. doi: 10.1002/DEV.420210404 3378678

[13] Vanderschuren LJMJ Achterberg EJM Trezza V . The neurobiology of social play and its rewarding value in rats. Neurosci Biobehav Rev. (2016) 70:86. doi: 10.1016/J.NEUBIOREV.2016.07.025 27587003 PMC5074863

[14] Parise LF Joseph Burnett C Russo SJ . Early life stress and altered social behaviors: a perspective across species. Neurosci Res. (2023) 211:65. doi: 10.1016/J.NEURES.2023.11.005 37992997 PMC11102940

[15] Siviy SM Panksepp J . In search of the neurobiological substrates for social playfulness in mammalian brains. Neurosci Biobehav Rev. (2011) 35:1821–30. doi: 10.1016/J.NEUBIOREV.2011.03.006 21414353

[16] Xiong Y Hong H Liu C Zhang YQ . Social isolation and the brain: effects and mechanisms. Mol Psychiatry. (2022) 28:191. doi: 10.1038/S41380-022-01835-W 36434053 PMC9702717

[17] Albers HE . The regulation of social recognition, social communication and aggression: vasopressin in the social behavior neural network. Horm Behav. (2012) 61:283–92. doi: 10.1016/J.YHBEH.2011.10.007 22079778

[18] Caldwell HK . Oxytocin and vasopressin: Powerful regulators of social behavior. Neuroscientist. (2017) 23(5):517–28. doi: 10.1177/1073858417708284 28492104

[19] Bielsky IF Young LJ . Oxytocin, vasopressin, and social recognition in mammals. Peptides. (2004) 25:1565–74. doi: 10.1016/j.peptides.2004.05.019 15374658

[20] Freeman SM Young LJ . Comparative perspectives on oxytocin and vasopressin receptor research in rodents and primates: Translational implications. J Neuroendocrinol. (2016) 28(4):10.1111/jne.12382. doi: 10.1111/jne.12382 26940141 PMC4886472

[21] Terranova JI Ferris CF Albers HE . Sex differences in the regulation of offensive aggression and dominance by arginine-vasopressin. Front Endocrinol. (2017) 8:308. doi: 10.3389/FENDO.2017.00308 29184535 PMC5694440

[22] Bielsky IF Hu SB Szegda KL Westphal H Young LJ . Profound impairment in social recognition and reduction in anxiety-like behavior in vasopressin V1a receptor knockout mice. Neuropsychopharmacology: Off Publ Am Coll Neuropsychopharmacol. (2004) 29:483–93. doi: 10.1038/SJ.NPP.1300360 14647484

[23] Bielsky IF Hu SB Ren X Terwilliger EF Young LJ . The V1a vasopressin receptor is necessary and sufficient for normal social recognition: A gene replacement study. Neuron. (2005) 47:503–13. doi: 10.1016/j.neuron.2005.06.031 16102534

[24] Keverne EB Curley JP . Vasopressin, oxytocin and social behaviour. Curr Opin Neurobiol. (2004) 14:777–83. doi: 10.1016/J.CONB.2004.10.006 15582383

[25] Albers HE Dean A Karom MC Smith D Huhman KL . Role of V1a vasopressin receptors in the control of aggression in Syrian hamsters. Brain Res. (2006) 1073–1074:425–30. doi: 10.1016/J.BRAINRES.2005.12.081 16445890

[26] Caldwell HK Lee HJ Macbeth AH Young WS . Vasopressin: Behavioral roles of an ‘original’ neuropeptide. Prog Neurobiol. (2008) 84(1):1–24. doi: 10.1016/j.pneurobio.2007.10.007 18053631 PMC2292122

[27] de Vries GJ Panzica GC . Sexual differentiation of central vasopressin and vasotocin systems in vertebrates: Different mechanisms, similar endpoints. Neuroscience. (2006) 138:947–55. doi: 10.1016/j.neuroscience.2005.07.050 16310321 PMC1457099

[28] Dumais KM Veenema AH . Vasopressin and oxytocin receptor systems in the brain: Sex differences and sex-specific regulation of social behavior. Front Neuroendocrinol. (2016) 40:1–23. doi: 10.1016/j.yfrne.2015.04.003 25951955 PMC4633405

[29] Brydges NM Hall J Best C Rule L Watkin H Drake AJ . Childhood stress impairs social function through AVP-dependent mechanisms. Transl Psychiatry. (2019) 9:330. doi: 10.1038/s41398-019-0678-0 PMC6901493 31819033

[30] Albers HE . Species, sex and individual differences in the Vasotocin/Vasopressin System: relationship to neurochemical signaling in the social behavior neural network. F Neuroendocrinol. (2014) 49. doi: 10.1016/J.YFRNE.2014.07.001 PMC4317378 25102443

[31] Song Z Larkin TE Malley MO Albers HE . Oxytocin (OT) and arginine-vasopressin (AVP) act on OT receptors and not AVP V1a receptors to enhance social recognition in adult Syrian hamsters (Mesocricetus auratus). Horm Behav. (2016) 81:20–7. doi: 10.1016/J.YHBEH.2016.02.004 26975586

[32] Taylor JH Walton JC McCann KE Norvelle A Liu Q Vander Velden JW . CRISPR-Cas9 editing of the arginine–vasopressin V1a receptor produces paradoxical changes in social behavior in Syrian hamsters. PNAS. (2022) 119(19):e2121037119. doi: 10.1073/pnas.2121037119 35512092 PMC9171636

[33] Chanut FJA Williams AM . The Syrian golden hamster estrous cycle: Unique characteristics, visual guide to staging, and comparison with the rat. Toxicol Pathol. (2016) 44(1):43–50. doi: 10.1177/0192623315607668 26516162

[34] National Research Council (US) Committee for the Update of the Guide for the Care and Use of Laboratory Animals . Guide for the Care and Use of Laboratory Animals. 8th ed. Washington, DC: National Academies Press (US) (2011). doi: 10.17226/12910 21595115

[35] Song Z McCann KE McNeill JK Larkin TE Huhman KL Albers HE . Oxytocin induces social communication by activating arginine-vasopressin V1a receptors and not oxytocin receptors. Psychoneuroendocrinology. (2014) 50:14–9. doi: 10.1016/J.PSYNEUEN.2014.08.005 25173438 PMC4252597

[36] Choleris E Gustafsson JÅ Korach KS Muglia LJ Pfaff DW Ogawa S . An estrogen-dependent four-gene micronet regulating social recognition: A study with oxytocin and estrogen receptor-α and -β knockout mice. PNAS. (2003) 100(10):6192–7. doi: 10.1073/pnas.0631699100 12730370 PMC156348

[37] Choleris E Ogawa S Kavaliers M Gustafsson J-A Korach KS Muglia LJ . Involvement of estrogen receptor alpha, beta and oxytocin in social discrimination: A detailed behavioral analysis with knockout female mice. Genes Brain Behav. (2006) 5:528–39. doi: 10.1111/j.1601-183X.2006.00203.x 17010099

[38] Dere E Huston JP De Souza Silva MA . The pharmacology, neuroanatomy and neurogenetics of one-trial object recognition in rodents. Neurosci Biobehav Rev. (2007) 31(5):673–704. doi: 10.1016/j.neubiorev.2007.01.005 17368764

[39] McCann KE Huhman KL . The effect of escapable versus inescapable social defeat on conditioned defeat and social recognition in Syrian hamsters. Physiol Behav. (2012) 105:493–7. doi: 10.1016/J.PHYSBEH.2011.09.009 21945371 PMC3225711

[40] McCann KE Bicknese CN Norvelle A Huhman KL . Effects of inescapable versus escapable social stress in Syrian hamsters: The importance of stressor duration versus escapability. Physiol Behav. (2014) 129:25–9. doi: 10.1016/J.PHYSBEH.2014.02.039 24582674 PMC4026280

[41] Blanchard RJ Yudko EB Rodgers RJ Blanchard DC . Defense system psychopharmacology: An ethological approach to the pharmacology of fear and anxiety. Behav Brain Res. (1993) 58:155–65. doi: 10.1016/0166-4328(93)90100-5 7907880

[42] Choleris E Thomas AW Kavaliers M Prato FS . A detailed ethological analysis of the mouse open field test: effects of diazepam, chlordiazepoxide and an extremely low frequency pulsed magnetic field. Neurosci Biobehav Rev. (2001) 25:235–60. doi: 10.1016/S0149-7634(01)00011-2 11378179

[43] Lister RG . Ethologically-based animal models of anxiety disorders. Pharmacol Ther. (1990) 46:321–40. doi: 10.1016/0163-7258(90)90021-S 2188266

[44] Cohen J . Statistical Power Analysis for the Behavioral Sciences. New York: Taylor and Francis (2013). doi: 10.4324/9780203771587

[45] Rigney N Zbib A de Vries GJ Petrulis A . Knockdown of sexually differentiated vasopressin expression in the bed nucleus of the stria terminalis reduces social and sexual behaviour in male, but not female, mice. J Neuroendocrinol. (2022) 34(9):e13083. doi: 10.1111/jne.13083 34978098 PMC9213575

[46] Aspesi D Choleris E . Neuroendocrine underpinning of social recognition in males and females. J Neuroendocrinol. (2021) 34(2):e13070. doi: 10.1111/jne.13070 34927288

[47] Rieger NS Guoynes CD Monari PK Hammond ER Malone CL Marler CA . Neuroendocrine mechanisms of aggression in rodents. Motivation Sci. (2022) 8:81–105. doi: 10.1037/MOT000026027371692

[48] Caldwell HK Lee HJ Macbeth AH Young WS . Vasopressin: behavioral roles of an "original" neuropeptide. Prog Neurobiol. (2008) 84(1):1–24. doi: 10.1016/j.pneurobio.2007.10.007 PMC2292122 18053631

[49] Rigney N De Vries GJ Petrulis A Young LJ . Oxytocin, vasopressin, and social behavior: From neural circuits to clinical opportunities. Endocrinology. (2022) 163:1–13. doi: 10.1210/ENDOCR/BQAC111 35863332 PMC9337272

[50] de Vries GJ Miller MA . Chapter 1.1 Anatomy and function of extrahypothalamic vasopressin systems in the brain. Progress in Brain Res. (1999) 119:3–20. doi: 10.1016/S0079-6123(08)61558-7 10074777

[51] Castaneda AJ Whitten CJ Menard TA Sisk CL Cooper MA Schulz KM . Testosterone differentially modulates the display of agonistic behavior and dominance over opponents before and after adolescence in male Syrian hamsters. Front Behav Neurosci. (2025) 19:1603862. doi: 10.3389/FNBEH.2025.1603862 40708844 PMC12287037

[52] Faruzzi AN Solomon MB Demas GE Huhman KL . Gonadal hormones modulate the display of submissive behavior in socially defeated female Syrian hamsters. Horm Behav. (2005) 47:569–75. doi: 10.1016/J.YHBEH.2004.11.023 15811359

[53] Solomon MB Karom MC Norvelle A Markham CA Erwin WD Huhman KL . Gonadal hormones modulate the display of conditioned defeat in male Syrian hamsters. Horm Behav. (2009) 56:423. doi: 10.1016/J.YHBEH.2009.07.011 19651128 PMC2762350

[54] Aspesi D Sambor E Stoehr MC Taylor J Grieb ZA Huhman KL . The deletion of the arginine vasopressin 1a receptor impairs sexual and maternal behavior. Front Endocrinol. (2025) 16:1649706. doi: 10.3389/fendo.2025.1649706 PMC12483901 41040855

[55] El-Brolosy MA Kontarakis Z Rossi A Kuenne C Günther S Fukuda N . Genetic compensation triggered by mutant mRNA degradation. Nature. (2019) 568:193. doi: 10.1038/S41586-019-1064-Z 30944477 PMC6707827

[56] Paletta P Aspesi D Bass N Choleris E . The study of social cognition: Social recognition and social learning in laboratory rats and mice. In: Paredes RG Portillo W Bedos M (eds.), Animal Models of Reproductive Behavior. New York, NY: Humana (2023). doi: 10.1007/978-1-0716-3234-5_1

[57] Shivakumar AB Mehak SF Jijimon F Gangadharan G . Extrahippocampal contributions to social memory: the role of septal nuclei. Biological Psychiatry. (2024) 96(11):835–47. doi: 10.1016/J.BIOPSYCH.2024.04.018 38718881

[58] Wang X Zhan Y . Regulation of Social Recognition Memory in the Hippocampal Circuits. Frontiers in Neural Circuits. (2022) 16:839931. doi: 10.3389/FNCIR.2022.839931 PMC9006871 35431817

[59] Chaouloff F . Social stress models in depression research: what do they tell us? Cell Tissue Res. (2013) 354:179. doi: 10.1007/S00441-013-1606-X 23532563 PMC4901160

[60] Rosenhauer AM Beach LQ Jeffress EC Thompson BM McCann KE Partrick KA . Brain-derived neurotrophic factor signaling mitigates the impact of acute social stress. Neuropharmacology. (2018) 148:40. doi: 10.1016/J.NEUROPHARM.2018.12.016 30557566 PMC6424638

[61] Bredewold R Veenema AH . Sex differences in the regulation of social and anxiety-related behaviors: insights from vasopressin and oxytocin brain systems. Curr Opin Neurobiol. (2018) 49:132–40. doi: 10.1016/j.conb.2018.02.011 29518698 PMC6055524

[62] Rigney N Vries GJ Petrulis A . Modulation of social behavior by distinct vasopressin sources. Front Endocrinol. (2023) 14. doi: 10.3389/FENDO.2023.1127792 36860367 PMC9968743

[63] Paul MJ Terranova JI Probst CK Murray EK Ismail NI de Vries GJ . Sexually dimorphic role for vasopressin in the development of social play. Front Behav Neurosci. (2014) 8:58. doi: 10.3389/fnbeh.2014.00058 PMC3937588 24616675

[64] Veenema AH Bredewold R De Vries GJ . Sex-specific modulation of juvenile social play by vasopressin. Psychoneuroendocrinology. (2013) 38:2554–61. doi: 10.1016/J.PSYNEUEN.2013.06.002 23838102 PMC3812261

[65] DiBenedictis BT Nussbaum ER Cheung HK Veenema AH . Quantitative mapping reveals age and sex differences in vasopressin, but not oxytocin, immunoreactivity in the rat social behavior neural network. J Comp Neurol. (2017) 525:2549. doi: 10.1002/CNE.24216 28340511 PMC6066795

[66] Hiura LC Ophir AG . Interactions of sex and early life social experiences at two developmental stages shape nonapeptide receptor profiles. Integr Zool. (2018) 13:745. doi: 10.1111/1749-4877.12338 29851289 PMC7412594

