# Supplementary material for: Nonlinear and sex-specific effects of V1aR deletion on juvenile social behavior
Source: Front Endocrinol (Lausanne). 2026 Sep 8;17:1901426. doi: 10.3389/fendo.2026.1901426 (PMC13593422; doi:10.3389/fendo.2026.1901426)
Supplement: Supplementary file 1 [file SupplementaryFile1.docx]

Supplementary

**Figure S1. Representative locomotor tracks during the social avoidance test**. Representative movement trajectories of wild-type (WT), heterozygous (Het), and knockout (KD) male and female hamsters during the 5-min social avoidance test. The gray box indicates the location of the unfamiliar resident aggressor (RA) confined within the holding box. Representative Het animals spent proportionally more time in the avoidance zone and less time near the resident aggressor than WT or KO animals, consistent with the quantitative analyses presented in Figure 5.
